# Renin–angiotensin system in human coronavirus pathogenesis

**DOI:** 10.2217/fvl.10.4

**Published:** 2010-03-01

**Authors:** Brigitte A Wevers, Lia van der Hoek

**Affiliations:** Center for Experimental & Molecular Medicine, Center for Infection & Immunity Amsterdam, Academic Medical Center, University of Amsterdam, PO Box 226600, 1100 DD Amsterdam, The Netherlands. b.a.wevers@amc.uva.nl; Laboratory of Experimental Virology, Department of Medical Microbiology, Center for Infection & Immunity Amsterdam, Academic Medical Center, University of Amsterdam, PO Box 226600, 1100 DD Amsterdam, The Netherlands. Tel.: +31 205 667 510; Fax: +31 206 916 531; c.m.vanderhoek@amc.uva.nl

**Keywords:** aminopeptidase N, angiotensin-converting enzyme 2, human coronavirus 229E, human coronavirus NL63, renin–angiotensin system, SARS-CoV, virus–host interactions

## Abstract

Although initially considered relatively harmless pathogens, human coronaviruses (HCoVs) are nowadays known to be associated with more severe clinical complications. Still, their precise pathogenic potential is largely unknown, particularly regarding the most recently identified species HCoV-NL63 and HCoV-HKU1. HCoVs need host cell proteins to successively establish infections. Proteases of the renin–angiotensin system serve as receptors needed for entry into target cells; this article describes the current knowledge on the involvement of this system in HCoV pathogenesis.

**Figure 1.**
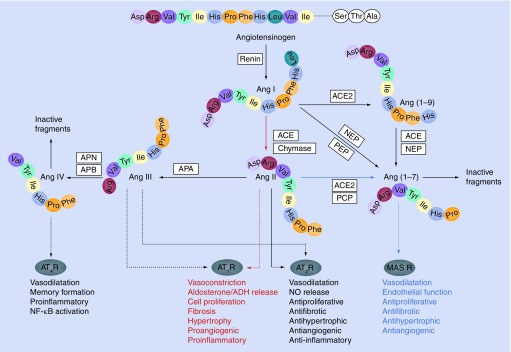
Overview of the most important renin–angiotensin components and mediated physiological effects. Linear enzymatic cascade depicted in the middle represents the classical renin–angiotensin system (RAS) and yields the effector peptide Ang II that can interact with AT_1_R or AT_2_R. The more complex RAS is capable of producing additional biologically active angiotensin fragments with independent actions, including Ang III, Ang (1–7) and Ang IV. Depicted colored pathways: (red) main ACE–Ang II–AT_1_ receptor axis, (blue) putative counter-regulatory arm of the RAS, involving ACE2–Ang (1–7)–MAS R. This dual function system is primarily driven by the ACE/ACE2 balance. ACE: Angiotensin-converting enzyme; ADH: Antidiuretic hormone or vasopressin; Ang: Angiotensin; APA: Aminopeptidase A; APB: Aminopeptidase B; APN: Aminopeptidase N; AT_1_R: Angiotensin II receptor type 1; AT_2_R: Angiotensin II receptor type 2; AT_4_R: Angiotensin receptor type 4; MASR: MAS receptor; NEP: Neprilysin or neutral endopeptidase; NF-κB: Nuclear factor-κB; NO: Nitric oxide; PCP: Prolyl carboxypeptidase; PEP: Prolyl carboxypeptidase.

Coronaviruses (CoVs) are enveloped viruses with a positive-strand RNA genome, which primarily target mucosal surfaces of respiratory and intestinal tracts in a wide range of mammals and birds to establish an infection [1,2]. For many years, the prototype human CoV (HCoV) isolates 229E and OC43 were causally linked to the ‘common cold’: mild and self-limiting infections of the upper respiratory tract [3,4]. The discovery of a previously unknown CoV as the etiological agent of SARS, SARS-CoV, which caused a global outbreak of acute and often severe atypical pneumonia in 2003, clearly demonstrated the pathogenic potential of CoVs in the human host [5–9]. Subsequent identification of two other novel HCoVs: HCoV-NL63 and HCoV-HKU1 [10–12], with the ability to cause serious lower respiratory tract complications, especially in weakened patients [13], further urged the need for a profound knowledge concerning their pathogenesis. With the exception of SARS [5], present knowledge regarding HCoV-induced pathologies is primarily based on data obtained from population-based studies. HCoV-NL63 and HCoV-HKU1 are considered common cold-causing pathogens in healthy adults, and are also associated with a more severe clinical spectrum of respiratory disease in young children, elderly and immunocompromised adults [4,14]. Detailed studies needed to prove a causal relationship with a specific pathology are hampered by the lack of a suitable animal model for both HCoV-NL63 and HCoV-HKU1 viruses and absence of a cell culture system to propagate HCoV-HKU1.

Viruses critically depend on host cell-encoded proteins and corresponding mechanisms to ensure their survival and replicative success. As a consequence, many host cell proteins are important contributors to the complex process of viral pathogenesis [15]. Cell surface components that are exploited as primary receptors to mediate viral entry represent the most obvious host cell proteins involved in establishment of a viral infection. Following target cell entry, several viruses are known to induce downmodulation of receptor expression. As a result, natural physiologic functions of these host cell components may be seriously impaired, with accompanying pathogenic consequences for infected cells, organ or individual. Paradoxically, viruses strongly benefit from downregulation of receptor expression [16], since it leads to controlled and productive infectious processes. Receptor downmodulation prevents infection of cells in which viral replication is already progressing [17], and is often needed to ensure efficient release of viral particles [18]. A number of viruses are known to induce cellular receptor modulation, including HIV, measles virus, influenza C virus and human herpes virus type 6 [19–22], as well as CoVs. Two integral proteases of the renin–angiotensin system (RAS), a major physiologic regulator of the cardiovascular system, facilitate cellular entry of several HCoVs: angiotensin-converting enzyme (ACE)2 and neutral aminopeptidase (aminopeptidase N [APN]) [23–26]. Here, we will discuss the interaction of SARS-CoV, HCoV-NL63 and HCoV-229E with renin–angiotensin proteases during their cellular entry, and the pathogenic consequences of HCoV-induced RAS dysregulation by receptor downmodulation at the primary site of infection.

## HCoV interactions with renin–angiotensin proteases

To establish and maintain an infection cycle, CoVs need to fuse their lipid bilayer envelopes with membranes of susceptible cells, and subsequently deliver their genetic material into the intracellular space. These crucial events are mediated by heavily glycosylated spike proteins present on the outer surface of the virion, which interact with appropriate host cell-surface entry structures [27]. Depending on the functional consequences of virus–host interactions, these surface molecules are of two general types. Attachment factors or co-receptors, often carbohydrate structures on the cell surface (i.e., lectins and sialic acids), probably only serve to bind coronaviral particles, and thus help to concentrate viruses on the cell surface. Often, interactions with attachment factors are not highly specific [15]. Unlike attachment factors, CoV receptors actively promote cell entry. They can do so by initiating conformational changes in the virus particle, activating signaling pathways, and promoting membrane fusion and internalization [15,28]. Although cellular receptors for HCoV-OC43 and HCoV-HKU1 remain to be elucidated, HCoV-229E, SARS-CoV and HCoV-NL63 strictly target membrane-associated proteases as their primary entry receptors.

Human coronavirus-229E, as well as related animal CoVs, including porcine-transmissible gastroenteritis virus, porcine respiratory CoV, feline infectious peritonitis virus, feline enteric CoV, canine CoV, and porcine epidemic diarrhea virus all use APN of their natural host as a functional receptor [26,29–31]. Human APN is a membrane-bound glycoprotein of 150 kDa with zinc-dependent protease activity [32], identical to the myeloid differentiation antigen, CD13, present on granulocytes, monocytes and their progenitors [33]. Structurally, APN is predicted to consist of a short amino-terminal intracellular fragment, a single transmembrane part and a large extracellular C-terminal region that holds the catalytic site [34]. Through its enzymatic activity, in which APN preferentially cleaves neutral amino acids from amino-terminal ends of oligopeptides, APN regulates activity of numerous biologically active peptides, including hormones, neuro- and vasoactive peptides, and cytokines [35]. APN participates in the removal of individual amino acids from peptides in lumen of the small intestine and processing of peptides bound to MHC-II molecules on antigen-presenting cells [36]. In addition, APN is believed to participate as a signaling molecule in angiogenesis, leukocyte adhesion and phagocytosis [35,37].

Aminopeptidase N exists as a heavily glycosylated homodimeric protease on the surface of a very broad range of cell types, including epithelial cells of kidney and intestine, endothelial cells, cells of the nervous system (i.e., cerebral peritocytes at blood–brain barrier and synaptic junctions), myeloid cells (monocytes as well as antigen-presenting cells) and fibroblasts [34–36]. APN is also localized to the apical surface of polarized respiratory epithelium, the site at which HCoV-229E infection starts [38]. In addition, pulmonary dendritic cells are an important source of APN expression and have been hypothesized to capture and transmit HCoV-229E from the respiratory epithelium to susceptible cells in other parts of the body, possibly contributing to viral spread during an infection [39]. Amino acids 260–353 from APN have been identified as an essential domain for binding HCoV-229E. Within this protein region, eight residues (i.e., amino acids 288–295) are critical for virus receptor activity [40]. This HCoV-229E binding region is not identical to the active site of APN and mutations in the catalytic domain do not affect CoV infection, implying that APN protease activity is not involved in CoV entry [41]. After binding APN, HCoV-229E presumably utilizes a caveolae-mediated internalization mechanism for host cell entry [42,43]. This pathway involves endocytosis via specialized lipid rafts (caveolea), plasma membrane localized microdomains, rich in cholesterol and sphingolipids, which serve to concentrate membrane-associated proteins (e.g., viral receptors) [44]. Notably, human APN is known to be a component of lipid rafts from various cell types [42,45,46]. Initially, human APN was also suspected to be the receptor for HCoV-NL63, since HCoV-NL63 S protein harbors most amino acid sequence similarity (56%) with the spike protein of HCoV-229E [10]. Nonetheless, ACE2 acts, quite surprisingly, as the functional HCoV-NL63 receptor [25].

Before its involvement in HCoV-NL63 infection was elucidated, the membrane-bound 120 kDa glycoprotein, ACE2, had been identified as a receptor for another HCoV: SARS-CoV [23,24]. A mouse SARS-CoV infection model and *ace2* knockout mice provided evidence that ACE2 is essential for natural SARS-CoV infections *in vivo* [24]. Furthermore, antibodies directed against ACE2 and soluble ACE2 molecules and derivatives were demonstrated to be capable of blocking a SARS-CoV infection [23,47]. Like APN, human ACE2 is a protease with a large catalytic site facing into the extracellular space, strictly dependent on zinc for its activity [48]. However, ACE2 is only distantly related to APN [49], and acts as a carboxypeptidase, cleaving solely single carboxy-terminal residues from a number of circulating and physiologically active substrates [50,51]. Although ACE2 shares 40% amino acid similarity with the ubiquitous metalloprotease ACE, this homolog is not implicated in HCoV entry [52]. ACE2 is expressed at apical plasma membranes of epithelial cells, including those of respiratory origin [53], the primary location of SARS-CoV and HCoV-NL63 infection [7,10]. Furthermore, ACE2 is abundantly expressed by cardiovascular endothelium, epithelial cells of the kidney and testis and, to a lesser extent, by small intestine epithelia [54,55]. Accordingly, SARS-CoV has been isolated from sites of the gastrointestinal tract, kidneys and human heart tissue [56,57]. A soluble and circulating form of the ACE2 ectodomain originates after proteolytic processing by TNF-α-converting enzyme (TACE or ADAM17) or disintegrin and metalloprotease 10 (ADAM10) [58,59]. Although this soluble form of ACE2 can be detected in plasma, urine and airway surface liquid, and is catalytically active [59–61], the clinical importance of this shedding process is still elusive [62].

While the spike proteins of SARS-CoV and HCoV-NL63 are rather different [63], according to their amino acid identity of only 14%, SARS-CoV and HCoV-NL63 bind to a common region of the ACE2 protein, which is localized on the outer N-terminal lobe and not implicated in ACE2 catalytic function [64,65]. Accumulating data are, however, indicative for a different host cell entry strategy for SARS-CoV and HCoV-NL63, in spite of targeting the same receptor. During endocytosis SARS-CoV requires the endosomal protease cathepsin L to initiate and maintain a successful infection, in addition to ACE2 binding [66,67]. Cathepsin L enzymes mediate cleavage and subsequent activation of membrane fusion activity of the SARS-CoV spike protein [68]. In marked contrast to SARS-CoV, HCoV-NL63 host cell entry occurs independently of cellular cathepsin activity [67], indicating the presence of an alternative and nonproteolytic strategy for initiation of the membrane fusion processes. Following attachment of ACE2, SARS-CoV spike proteins efficiently induce TACE-dependent proteolytic release of catalytically active ACE2 ectodomains, a shedding process also coupled to induction of TNF-α production [69,70].

Besides their classification as zinc-dependent peptidases, APN and ACE2 share another important functional characteristic. Both proteins are integral components of the RAS, one of the most important regulators of human physiology. SARS-CoV-induced dysregulation of the RAS is currently postulated to contribute to generation of severe clinical signs observed during SARS-CoV infections [24]. During establishment of an infection, SARS-CoV induces a rapid downregulation of ACE2 cell surface expression, either via internalization of the receptor–ligand complex [71,72] or activation of TACE-mediated ectodomain shedding of soluble ACE2 [59,69,70], which is proposed to seriously alter physiologic functionalities of pulmonary RAS. Unpublished data from our laboratory demonstrates that HCoV-NL63 infection induces a reduction of cellular ACE2 expression [Dijkman R, van der Hoek L, Unpublished Data], but the rate at which the downregulation of ACE2 occurs is probably delayed (less than 4 days after infection of LLC-MK2 cells). Other studies did not observe HCoV-NL63-induced ACE2 downregulation, but these studies either measured during a very short period (12 h) [69] or had only low-level HCoV-NL63 infection (less than 2 log rise in viral RNA load) [70]. Shedding of ACE2 following HCoV-NL63 infection has also been noted, but the concentration of cleaved, soluble ACE2 was higher during SARS-CoV than HCoV-NL63 infection [70].

Aminopeptidase N is targeted as an entry receptor by HCoV-229E. Following binding of HCoV-229E, APN aggregates and translocates to caveolin-enriched membrane domains, leading to endocytosis and virus internalization [42]. Receptor-mediated endocytosis of viral particles generally results in simultaneous internalization of the entry receptor [28,73]. Indeed, sequestration of porcine APN molecules into intracellular vesicles has been visualized during endocytosis of porcine CoV strain transmissible gastroenteritis virus [74]. HCoV-229E-induced downregulation of APN expression is likely to occur, yet strong evidence is currently lacking. Similar to ACE2 downmodulation, HCoV-229E-induced abrogation of cellular APN expression may impair its normal physiological function within the RAS and contribute to development of clinical symptoms, raising the interesting possibility that the capacity of several HCoVs to provoke clinical manifestations might be (in part) explained by specific interactions with the RAS.

## RAS: regulating human physiology

The RAS is one of the best described endocrine systems, playing a key role in maintenance of arterial pressure, fluid homeostasis, salt balances and cardiac function. Moreover, RAS classically regulates tissue remodeling processes, in particular cell proliferation, hypertrophy, angiogenesis and apoptosis [75]. In addition to its involvement in normal physiological processes, the system has been connected to numerous pathophysiological processes [76]. Abnormal activated RAS has well established roles in development of cardiovascular diseases (ranging from hypertension to heart failure), renal diseases and diabetes [77–79]. Within the RAS, various angiotensin peptides are synthesized and degraded from the large precursor angiotensinogen, by means of a complex series of enzymatic reactions (Figure 1). While some RAS components are generated at specific sites of the body (e.g., renin from kidneys, lung-derived ACE and angiotensinogen from the liver), actual angiotensin synthesis is believed to occur in every organ [80].

Renin–angiotensin system activity is initiated by the kidney, through release of renin from juxtaglomerular cells [81]. Renin is an aspartic protease that enzymatically cleaves its substrate angiotensinogen, which is produced by the liver, to form an inactive peptide: angiotensin (Ang)I or Ang (1–10). Ang I is subsequently converted into the major RAS effector peptide Ang II or Ang (1–8), through activity of the zinc-dependent protease ACE, which hydrolyzes two amino acids from the carboxy terminus of Ang I [82]. ACE is expressed in high concentrations on surfaces of vascular endothelial cells, particularly in lung tissue [75]. Moreover, Ang II can be generated from Ang I through the activity of non-ACE-related enzymes, including the serine protease chymase [83]. Although ACE is regarded as the primary Ang II-converting enzyme, in certain vascular pathological conditions the majority of Ang II is most likely generated by chymase [84]. Moreover, in pulmonary membranes, chymase activity has been detected, making an import contribution to the conversion of Ang I into Ang II in lung tissue [85]. Ang II initiates most of the RAS-attributed physiologic effects through selective interactions with G-protein-coupled Ang II type 1 (AT_1_) or type 2 (AT_2_) receptors and subsequent activation of distinct intracellular signaling pathways [84,86]. In principle, activated AT_1_ and AT_2_ receptors mediate opposing functions. AT_1_ receptors predominantly orchestrate most of the well-known and classical physiological actions of Ang II, and are abundantly expressed in a variety of organs, including liver, kidney, brain, lung, heart and vascular system [87]. Activated AT_1_ receptors are able to regulate arterial pressure through vasoconstriction, fluid and sodium balance by means of activation of antidiuretic hormone and aldosterone secretion, fibrosis, cellular growth and migration [75,84]. More recently, Ang II binding to AT_1_ receptors has been implicated in inflammatory responses [88–91]. When improperly counterbalanced, AT_1_ receptors might induce potentially harmful actions and contribute to pathogenesis. Activation of this Ang II–AT_1_ receptor-dependent pathway is widely accepted to lead to organ damage and fibrosis [84]. In contrast to the extensively characterized AT_1_ receptor responses, physiological consequences of AT_2_ receptor activation are still not completely unraveled. AT_2_ receptor activation by RAS components is generally assumed to result in more protective clinical consequences, partially by counteracting AT_1_ receptor responses [92,93]. AT_2_ receptor stimulation has been associated, for instance, with protection of the brain against ischemia [94]. In essence, AT_2_ receptors are linked to vasodilatation, release of nitric oxide, tissue development and remodeling, by stimulating apoptosis and inhibition of cell growth [95].

## Emerging aspects of the RAS

The aforementioned enzymatic reactions, which lead to Ang II formation, together constitute the ‘classical’ or ‘renal’ RAS cascade, initially described in 1940 (depicted in the middle of Figure 1) [96]. Ever since, additional RAS components have been discovered that make this classical pathway more complex than previously thought. Clearly, Ang II is not a unique RAS end product and the multiple-mediator system operates far beyond relative simple regulation of fluid and blood pressure homeostasis [75,97–99].

Angiotensin-converting enzyme has been regarded as the key regulating enzyme within the RAS for a long time [100,101]. Currently, it is well established that various additional enzymes target both Ang I and II to induce formation of alternative angiotensin peptide fragments, in particular Ang (1–7), Ang III or Ang (2–8) and Ang IV or Ang (3–8) [84]. These proteins are proven to function as unique, physiologically active RAS components, although initially identified as inactive breakdown products. Ang (1–7), Ang III and IV exert their own specific biological actions via individual receptors, and are, in addition, believed to modulate the classical effects induced by Ang II. The Ang I-derived heptapeptide Ang (1–7) can be generated directly through action of several tissue endopeptidases, including neutral endopeptidase (or neprilysin) and prolyl endopeptidase [102]. After discovery of a novel RAS enzyme homologous to ACE (the carboxypeptidase ACE2) [48,49], two additional pathways leading to Ang (1–7) formation were introduced. At first, ACE2 has been demonstrated to induce conversion of Ang I into Ang (1–7) by means of intermediate production of Ang (1–9), a fragment with unknown function [49,98]. Ang (1–9) is subsequently further metabolized into Ang (1–7) through enzymatic activity of ACE [103]. Second, ACE2 is able to hydrolyze Ang (1–7) directly from Ang II [104]. Of these two ACE2-mediated enzymatic steps, direct conversion of Ang II into Ang (1–7) is kinetically most favorable [50,105]. Alternatively, immediate generation of Ang (1–7) from Ang II can be orchestrated through activity of several other enzymes, including prolyl carboxypeptidase [106], which is predominantly expressed in vascular endothelial cells [102]. However, ACE2 is by far the most potent enzyme in hydrolyzing Ang II into Ang (1–7) [50,107]. Once synthesized, Ang (1–7) exerts its functions through association with the G-protein-coupled receptor Mas (MAS R), initially misidentified as an Ang II receptor [98,108]. There is, however, also evidence supporting MAS R-independent Ang (1–7) activity [109]. Ang (1–7) induces multiple actions (i.e., vasodilatation and cellular growth inhibition) that appear to counterbalance most of the physiologic responses initiated by Ang II [82,105,110].

The ACE2–Ang (1–7)–MAS R axis is regarded as a putative ‘pressor’ or counter-regulatory arm of the RAS, producing effects that oppose those of the main ACE–Ang II–AT_1_ receptor axis (Figure 1; blue and red pathways, respectively). In this concept, RAS is envisioned mainly as a dual function system, in which actions (i.e., vasoconstriction/vasodilatation and proliferative/antiproliferative) are primarily driven by the ACE/ACE2 balance [78,102,111]. A higher level of ACE will lead to an increased Ang II generation and decreased catabolism of Ang (1–7), while an opposite ratio (i.e., higher ACE2 levels) will contribute to reduced Ang II and elevated Ang (1–7) levels. Ang (1–7) that directly antagonizes many of the Ang II-mediated actions and provides an additional level of counter regulation [111]. Genetic ACE2 inactivation experiments confirmed an important role for ACE2 in regulating Ang II levels *in vivo*. To date, all strains of *ace2* knockout mice are reported to possess increased plasma and tissue levels of Ang II [112–114].

Besides formation of Ang (1–7), Ang II can be degraded further into two distinct bioactive peptides (i.e., Ang III and Ang IV) through activity of several membrane-bound aminopeptidases [92,115], which are still confusingly identified by several different names (Box 1). Ang III is readily synthesized from Ang II through enzymatic involvement of aminopeptidase A, which hydrolyzes acidic amino acids [116,117]. Similar to Ang II, Ang III orchestrates its physiologic functions through binding and activation of AT_1_ and AT_2_ receptors [92,98]. Ang III is, however, less potent compared with Ang II in mediating biological responses via these receptors [115]. Most likely, this is attributable to the high catabolic rate of Ang III formation, which occurs three-times faster than that of Ang II due to broad peripheral distribution of APN [118]. Like Ang II, Ang III participates in cardiovascular and renal functions, stimulating production of aldosterone and decreasing renal blood flow and renin secretion [84,92,119]. In specific AT_1_-mediated actions Ang III might be even more important than Ang II (e.g., vasopressin release in the brain) [84]. Ang III also plays a major role in brain physiology, regulating water homeostasis, blood pressure and norepinephrine release [119].

Angiotensin III can be degraded into a third alternative component, designated Ang IV, through action of two additional aminopeptidases: APN and aminopeptidase B (APB) [98,120,121]. Whereas APN cleaves neutral amino acids, APB preferentially hydrolyzes basic residues from the N-terminal side of proteins [116]. The affinity of Ang IV to bind classical RAS receptors AT_1_ and AT_2_ is very low [92,120]. Instead, Ang IV possesses the potential to bind and signal through the recently identified angiotensin type 4 (AT_4_) receptor [120,122]. Ang IV is linked to several important physiologic functions, including blood flow regulation, processes underlying learning and memory formation, and activation of proinflammatory genes [123–125]. This Ang IV-mediated activation of transcription factor NF-κB leads to increased expression of platelet activator inhibitor-I, monocyte chemoattractant protein-1, IL-6 and TNF-α [84,126].

## Local RAS at primary site of HCoV infections

Traditionally, the RAS has been described predominantly as a circulating and endocrine system. It is now well established that besides a peripheral RAS, tissue-specific or local systems exist as well. In many organ systems, including the heart, brain, lung, kidney, pancreas, liver and vasculature, as well as the nervous, reproductive and digestive systems, RAS components necessary for biosynthesis of active angiotensin fragments have been detected [82]. As a result, these organs are thought to possess the capacity to generate RAS effector proteins locally. Tissue-specific systems exert diverse paracrine and/or autocrine mechanisms, which have been functionally correlated to several cell-specific effects, including cell growth, proliferation and metabolism [82]. While independent operation of tissue RAS is reported in some organs (e.g., the brain), other systems (from heart and kidney) function in close cooperation with circulating RAS [84].

Although speculated for a long time, actual existence of a local airway RAS and the capacity to generate intrapulmonary Ang II has been confirmed only recently [127]. The lungs have been demonstrated to possess a local system that is not driven by kidney-derived renin from the circulation. Instead, alveolar mast cells, which populate the upper and lower respiratory tract, express renin, which triggers pulmonary Ang II formation [127–129]. Other classical components of the RAS have been detected abundantly in rodent and human airway tissue [130,131], including angiotensinogen and ACE, the pulmonary epithelium being the primary source for circulatory ACE [132]. Ang II receptors are expressed in lungs as well, with the AT_1_ subtype found in bronchial smooth muscle cells, and AT_2_ receptors detected at bronchial epithelial brush borders [133]. ACE2 expression has been confirmed in alveolar and bronchiolar epithelial cells, as well as pulmonary endothelial cells [54]. Chymase is present in lung mast cells as well, and may be a major Ang II-generating enzyme in the lung [85]. Local pulmonary RAS is shown to contribute to several tissue remodeling processes, including regulation of alveolar epithelial cell apoptosis, enhancement of fibroblast proliferation and lung collagen production [134,135]. However, inappropriate activation and regulation of local airway renin–angiotensin components, in particular ACE2, might also initiate development of lung-associated pathophysiological conditions.

## ACE2 & its role in acute lung injury

Angiotensin-converting enzyme 2 is thought to be a key player in maintenance of RAS homeostasis, preferably through its ability to convert Ang II into Ang (1–7), and to activate the putative ‘pressor’ pathway within the RAS (blue pathway in Figure 1). By doing so, ACE2 may well be able to regulate the net level of Ang II present in tissues, and to antagonize hypertrophic and fibrotic effects resulting from Ang II binding to the AT_1_ receptor (red pathway in Figure 1) [99,102]. This counter-regulatory role of ACE2 might also be crucial in maintenance of lung RAS homeostasis [99,136]. Improperly regulated and increased ACE and Ang II levels have been specifically associated with pathogenesis of different forms of lung diseases, including pulmonary hypertension, sarcoidosis, pulmonary fibrosis and acute respiratory stress syndrome (ARDS) [137–140]. Most notably, *in vivo* studies confirmed a functional association between pulmonary RAS and ARDS severity and outcome [141]. ARDS is regarded as the most severe form of acute lung injury, characterized by pulmonary edema, accumulation of inflammatory cells and severe hypoxia [142]. Multiple pathogenic conditions can trigger development of ARDS, including sepsis, gastric juice aspiration, pancreatitis and trauma [143]. Several studies in the past have been pointing towards the importance of pulmonary RAS during the pathogenesis of this type of acute lung injury. Increased levels of ACE are detected in bronchoalveolar fluid of individuals suffering from ARDS [140]. Furthermore, ACE antagonists (i.e., AT_1_ receptor blockers) have been demonstrated to delay the onset of ARDS in a rat model with acute lung injury [144]. ACE is thought to negatively contribute to ARDS pathogenesis via a number of mechanisms, including an increase in vascular permeability and reduction of pneumocyte survival [145].

A recent study performed with an ARDS mouse model established an opposing and protective role for the pulmonary RAS component ACE2 [141]. Namely, mutant mice with an abrogated ACE2 expression developed a more severe disease pattern after acid aspiration-induced acute lung injury compared with control mice. These *ace2* knockout mice exhibited enhanced vascular permeability, increased lung edema, neutrophil accumulation and a worsened overall lung function. Importantly, systemic treatment of both knockout and wild-type mice with recombinant ACE2 improved ARDS symptoms. ACE and AT_1_ receptor, on the contrary, are negatively involved in ARDS pathogenesis, since loss of ACE or AT_1_ receptor expression correlates to a far less severe ARDS phenotype. Thus, ACE2 protects mice from severe acute lung injury/ARDS, preferably by negative regulation of Ang II levels. As opposed to ACE2, several other RAS components, including Ang II, ACE and AT_1_ receptor elevate disease pathogenesis, induce lung edemas and impair lung function [141]. Apparently, enzymes that may facilitate alternative Ang II degradation and subsequent production of Ang (1–7) [106,146], are not sufficient to protect against deterioration of lung injury by neutralizing an abrogated ACE2 expression. Indeed, prolyl carboxypeptidase and prolyl endopeptidase are known to be unspecific in their enzymatic activity, and possess a 10 –600-fold lower catalytic efficiency compared with ACE2 for the generation of Ang (1–7) [107]. Still, absence or inadequate expression of both these Ang II-converting enzymes in the pulmonary system might explain a lack of putative counter-regulatory pathways.

The major role of pulmonary RAS during lung failure is emphasized further by clinical studies performed in humans. A significant association between an ACE insertion/deletion polymorphism that affects ACE activity and susceptibility and outcome in ARDS has been found [147,148]. First, it has been demonstrated that ARDS patients carrying the insertion/insertion genotype, associated with lower ACE activity, have a survival rate that is significantly increased [148]. Second, the deletion/deletion allele has been observed significantly more in ARDS patients compared with a control group [147]. Most intriguingly, the discovery of ACE2-mediated protection in acute lung injury/ARDS simultaneously provided a mechanistic explanation for SARS-CoV pathogenesis [24].

## Role of the RAS in SARS-CoV & HCoV-NL63 infection

The high mortality rate (approaching 10%) following the SARS-CoV epidemic in 2003 is primarily attributable to respiratory failure caused by development of ARDS [136,149]. To infect its target host cells, SARS-CoV utilizes ACE2, the RAS component now known to orchestrate protection from acute lung failure/ARDS [23,24,150]. Notably, after engagement of viral spike proteins with ACE2, the amount of cell surface-expressed ACE2 is reduced [24,151]. This phenomenon of ACE2 receptor downregulation has been shown to provoke a worsening of lung failure in a SARS-CoV-infected mouse model [145]. By possessing a remarkable higher level of systemic Ang II, SARS-CoV-treated wild-type mice resemble the phenotype observed in *ace2* knockout mice, once again emphasizing the key role of ACE2 during ARDS [150]. The worsened ARDS symptoms observed in infected mice could be partially reversed by AT_1_ receptor blocker treatment, proving that continuous Ang II binding to AT_1_ receptors promotes exacerbation of lung injury during SARS-CoV infection [24]. Thus, SARS-CoV can deteriorate acute lung failure through dysregulation of pulmonary RAS activities. It is worth noting that studies analyzing the role of human *ace2* gene polymorphisms in progression of lung injury during SARS-CoV infections did not confirm such a correlation [152,153]. Other unknown factors are possibly involved in the overall mechanism of lung damage induced by SARS-CoV [154].

At present, there are some indications that downregulation of myocardial ACE2 expression by SARS-CoV induces symptoms of cardiac damage and dysfunction in some SARS patients, including arrhythmias, sudden cardiac death and systolic as well as diastolic dysfunction. Similarly to respiratory infection, SARS-CoV infection of the heart induces a downregulation of cellular ACE2 expression [57]. As an essential regulator of heart function, ACE2 has well established roles in the development of cardiovascular diseases [77]. A reduction in cardiac ACE2 expression most likely results in locally elevated levels of Ang II and might, therefore, also account for these pathophysiological processes, by Ang II-mediated activation of AT_1_ receptors and loss of the protective effects of Ang (1–7) [57,112]. However, direct evidence for this hypothesis is still lacking.

Although a possible molecular explanation for the severe clinical outcome during SARS-CoV infections is now available, questions remain about the precise pathogenic mechanisms of HCoV-NL63, which also utilizes ACE2 to infect human respiratory cells [25]. In contrast to SARS-CoV, clinical symptoms during HCoV-NL63 infections are usually mild to moderate and alveolar damage is rarely seen. More severe respiratory disease is only observed in immunocompromised patients, the elderly and children [4]. Several scenarios might explain this discrepancy of ACE2 utilization with absence of ARDS symptoms. As discussed, this might be, in part, caused by different cell entry strategies of both CoVs after ACE2 binding [151]. Furthermore, varying binding efficiency to ACE2 could be a feasible explanation [25]. HCoV-NL63 seems to bind ACE2 with a lower affinity compared with SARS-CoV [70,155,156]. Moreover, HCoV-NL63 might lack a pathogenicity factor that is present in SARS-CoV. This factor could possibly be encoded by an accessory gene of the SARS-CoV genome [25]. While SARS-CoV possesses an unusually high number of these genes (eight) with a still unknown function, only one is detected in the HCoV-NL63 genome [14,157].

The performance of the RAS is well known to decrease during normal aging processes, exemplified by a progressive decrease in circulating renin and plasma renin activity [158,159]. In this respect, it is important to note that a recent study in post-mortem lung tissue of deaths attributed to chest infection or pneumonia described a high percentage of HCoV-NL63 infection (5.3%), most of whom were elderly [160], indicating that with increasing age, HCoV-NL63 might lead to significant mortality. To date, only one HCoV-NL63-related death has been described in the literature, namely a 92-year-old man [161]. Aging seems to be involved in SARS-CoV infections as well, since the strongest predictor of poor disease outcome appears to be an advanced age (>60 years) [162]. Thus, a HCoV infection may cause more RAS-related damage in the elderly because of an impaired overall RAS activity.

## Involvement of the RAS in HCoV-229E infection

Aminopeptidase N, which converts Ang III into Ang IV within the RAS cascade (Figure 1), functions as a receptor for HCoV-229E [26]. Similar to ACE2 downregulation, an abrogation of APN expression could also give rise to potential pathophysiological consequences. Unfortunately, knowledge about APN and its putative role in HCoV-229E pathogenesis is insufficient and far from complete. This is probably, in part, attributable to the absence of severe symptoms during HCoV-229E infections in healthy adults, which are generally associated with mild and self-limiting upper respiratory tract diseases or ‘common colds’ [4,163]. An increasing body of evidence is, however, pointing towards more severe upper and lower respiratory tract illnesses, such as pneumonia, in young children, elderly and immunocompromised individuals [4]. Moreover, certain studies advocate for a putative neuroinvasive and neurovirulent capacity for HCoV-229E [164,165]. Unraveling a possible contribution of RAS in HCoV-229E infections, in particular through functioning of its enzymatic component APN, might therefore still provide important novel insights in pathogenic mechanisms of this human pathogen. Putative physiological consequences after APN-mediated RAS deregulation might be explained by several distinct scenarios. Here, some of the most interesting theories are discussed, with an emphasis on possible mechanisms evoking or dampening respiratory tract pathologies.

### Increased levels of Ang III

Within the RAS, APN is involved in conversion of Ang III to Ang IV [98,120]. This process can be blocked using a specific APN inhibitor (PC-18), which increases the half-life of Ang III by 3.9-fold [166]. Similarly, APN dowregulation during HCoV-229E infections will most probably contribute to an elevated Ang III expression level at sites of HCoV-229E infection. It is tempting to speculate that, like Ang II upregulation, increased Ang III levels might imbalance RAS and subsequently initiate pathological processes. Most of the current knowledge involving RAS-mediated harmful processes, however, only provide specific evidence for Ang II as a key player and not much is demonstrated for Ang III thus far. Ang II is considered to be a growth factor that regulates cell proliferation/apoptosis and fibrosis, as well as a proinflammatory mediator that attracts inflammatory cells to sites of tissue injury [167]. Since it shares many of its physiological properties with Ang II [92,98], Ang III is currently postulated to participate in certain harmful processes as well. In particular, Ang III has been reported to participate in initiation and progression of kidney injury [168]. In mesangial and renal interstitial fibroblast, Ang III binding to AT_1_ receptors is associated with overexpression of growth-related, profibrotic and proinflammatory genes, a prominent one being TGF-β [169]. TGF-β is known to be a key player in development of morphological alterations, including fibrosis and atrophy, by stimulating synthesis of extracellular matrix components and reducing collagenase production [168,170]. Also, at sites of human lung tissue injury, TGF-β expression is detected and inhibition of TGF-β in animal models attenuates development of fibrosis [171]. In contrast to a possible role for Ang III overexpression in kidney fibrotic processes, lung tissue injury exacerbation is assumed to be predominantly controlled by Ang II, again by activation of AT_1_ receptors [172–174]. Ang II is thought to exert its fibrotic effects by inducing TGF-β1 production, and triggering fibroblast proliferation and differentiation into collagen-secreting cells [175]. AT_1_ receptor binding by Ang III might be an organ-specific event and in some organs, including the pulmonary system, Ang II is more important in activating AT_1_ receptors. Results of several studies are indeed pointing towards Ang II as a main effector peptide in lung tissue. Locally produced Ang II in the lung, for example, has recently been demonstrated to be a critical factor in governing bronchial smooth muscle contraction through activation of AT_1_ receptors [127]. Moreover, Ang II is involved in exacerbation of acute lung injury [141]. However, research regarding Ang III function is still in its infancy and a putative role for Ang III-mediated pathophysiological processes in the respiratory tract is currently unknown and yet to be defined. As a consequence, a role for Ang III in HCoV-229E pathogenesis is far from confirmed.

### Reduced levels of Ang IV

Besides a putative elevated expression of Ang III, downregulation of APN may equally result in a reduced formation of Ang IV proteins. In this scenario, AT_4_ receptors are insufficiently activated. Presence of Ang IV-specific binding sites has been identified in various tissues and cells, including brain, spinal cord, colon, heart, kidney and vascular endothelial cells [176,177]. With regard to the pulmonary system, Ang IV plays a role in the regulation of blood flow by stimulating release of nitric oxide, a potent vasoconstrictor [178]. Furthermore, Ang IV seems to contribute to proliferation of lung vascular endothelial cells [177]. Most likely, these effects are attributable to systemic Ang IV, which is produced by the ‘peripheral RAS’ and released into the vascular system. Data regarding local pulmonary production of Ang IV and AT_4_ receptor expression are unfortunately unavailable. Therefore, uncertainty remains as to what extent Ang IV is an actual pulmonary RAS effector peptide, and whether it could be involved in HCoV-229E pathogenesis.

### APN-null mice

With respect to *apn* knockout mice, nothing relevant for the mechanism of HCoV-229E pathogenesis has been reported thus far [179]. Despite the broad range of APN functions, mice deficient in APN expression are not severely attenuated, and support only a role for APN in regulation of arterial blood pressure and the pathogenesis of hypertension [179,180]. This does certainly not exclude a role for APN in HCoV-229E pathogenesis, since initial investigations regarding *ace2* ^-/-^ mice also did not reveal the present knowledge regarding ACE2 and acute lung injury exacerbation [112–114]. As a consequence, simultaneous induction of acute lung injury/ARDS in this animal model is necessary to elucidate the actual involvement of pulmonary APN in HCoV-229E pathogenesis.

### Beneficial effects

In fact, severe clinical symptoms are generally not observed during HCoV-229E infection and this does not correspond to the previously described potential harmful consequences following modulation of APN expression. With respect to this, an attenuated APN expression at sites of HCoV-229E entrance might also give rise to effects that are beneficial for the host. Most striking is the recent suggestion that APN might actually be involved in regulation of chronic inflammation in the lung, by initiating chemokine production [181]. A continuous influx of inflammatory cells into the lung is not always beneficial for the host and might initiate fibrotic processes and organ-damaging processes. Systemic application of a specific APN inhibitor (actinonin) in a silica-induced murine model of lung fibrosis reduced chemokine secretion (e.g., IL-6 and monocyte chemoattractant protein-1) in lung and bronchoalveolar lavage fluid, and resulted in a decreased level of pulmonary fibrosis [181]. Other systemic effects during these APN inhibitor treatment experiments were not observed. In addition, reduced chemokine secretion after APN inhibitor treatment has been confirmed *in vitro*, in cultured human lung epithelial cells, advocating for an important role for APN in orchestrating pulmonary chemokine production [181]. A putative involvement of APN in chronic lung inflammation has also been suggested by another study. The activity of APN in bronchoalveolar lavage fluid was significantly higher in patients with sarcoidosis compared with control individuals, correlating to the number of infiltrating T lymphocytes [182]. APN is therefore thought to participate in inflammatory processes by orchestrating lymphocyte chemotaxis [35].

The rapid accumulation of proinflammatory cytokines (hypercytokinemia or ‘cytokine storm’) and chemokines in the respiratory tract is regarded as a prominent mediator in the pathogenesis of viral infections [183]. Concentrations of IL-6 and -8 in nasal secretions, for instance, correlate with the severity of symptoms observed during upper respiratory tract infections [184,185]. SARS-CoV, murine hepatitis virus and feline infectious peritonitis virus infections are also characterized by excessive and local invasion of activated immune cells and cytokines [9,162,186]. Therefore, it is interesting to speculate that APN is a contributor in the host response against coronaviral infections of nasal mucosa, and that its downregulation might also be accompanied by a dampening of disease pathogenesis. On the contrary, a stark reduction of cytokine and chemokine production is certainly not at all beneficial for the host and will give rise to serious adverse effects, including failure to efficiently combat the viral infection. Maintenance of a balance between host defenses and respiratory tract injury (e.g., fibrosis) is therefore essential [187].

It is debatable to what extent these putative APN-mediated inflammatory effects (reduction of chemokine production and lymphocyte chemotaxis) are attributable to an imbalance within pulmonary RAS pathways, since a definite mechanistic explanation is not provided by these preliminary studies. Hypothetically, APN may exert its inflammatory potential through formation of the RAS effector peptide Ang IV (Figure 1). On the contrary, it might also be a result of initiation of intracellular signaling and subsequent gene activation by APN, a RAS-independent activity [35]. When APN-mediated intracellular signaling mechanisms are indeed essential in these putative lung protective processes, triggering of such cascades upon HCoV-229E binding should happen as well. It is unknown whether HCoV-229E possesses the capacity to activate intracellular signaling pathways after association with APN [35]. Nonetheless, an abolished APN expression level seems to attenuate development of chronic lung inflammation and pulmonary fibrosis, which is in direct contrast to ACE2 downregulation, which results in exacerbation of lung disease [141]. Notwithstanding the fact that more evidence is definitely needed to demonstrate this APN-induced chemokine production and enhancement of pulmonary disease pathogenesis, it might provide an explanation for the lack of severe clinical symptoms during most HCoV-229E infections. A reduced level of APN expression following HCoV-229E binding would in fact decrease the amount of pulmonary damage after initiation of infection. However, this theory encompassing a major role of APN in pulmonary inflammation might very well be over simplistic since inflammatory responses are generally very complex.

## RAS as a highly dynamic system

Besides a potential beneficial effect of downregulation of APN expression, there are several alternative explanations for the general absence of severe clinical complications during HCoV-229E infections. APN is not unique in its ability to convert Ang III into Ang IV. APB is involved in this process as well [98,121]. This raises the possibility that APB might compensate for an abolished activity of APN, at the same time implicating that Ang III degradation is not severely attenuated. This theory is supported by the notion that RAS is a highly dynamic and multilayered system [84]. For instance, genetically engineered mice that do not express endothelial ACE and therefore lack ACE within the lung, are still capable of maintaining normal physiology. Lung chymase or an increased generation of Ang II by nonendothelial ACE may counterbalance the lack of local ACE expression, revealing a compensatory mechanism within the RAS [188]. It is, therefore, not unlikely that besides APB, additional bypass mechanisms exist that maintain normal physiology after virus-induced APN internalization.

## Conclusion

Increasing evidence is pointing towards an important role for the RAS during HCoV pathogenesis. Here, we addressed the specific interplay of three HCoVs with the RAS, in order to obtain novel clues regarding their pathogenicity in the human host. Clearly, pulmonary RAS negatively affects severe acute lung injury during SARS-CoV infection. Nevertheless, a lot of issues regarding ACE2 involvement in CoV pathogenesis still remain unsolved. Particularly, the lack of severe lung injury after infection with the newly identified HCoV-NL63, which also targets ACE2 as its primary entry receptor, is remarkable. The absence of a compensatory mechanism within RAS for the abolished pulmonary ACE2 expression is also highly interesting and will require further investigation.

Although far from confirmed, an interesting and putative role for a second RAS component, APN, in HCoV infection is reviewed as well. Preliminary data provide an attractive explanation for the general absence of severe clinical symptoms during infections with HCoV-229E. Disturbances in APN expression levels might protect from an overactivated inflammatory response (‘cytokine storm’), a process linked to initiation of organ fibrosis and failure, by orchestrating lymphocyte chemotaxis into HCoV-229E-infected lung tissue. Important clues might be obtained by lung injury induction studies with animal models lacking APN expression, since current knowledge is strictly based on APN inhibitor experiments.

Coronaviral pathogenesis is a highly complex process in which interactions between host and pathogen determine the outcome of virus-induced disease. It is becoming evident that the RAS is involved in HCoV infections, and presumably of high importance for their pathogenicity. In addition, these findings once more confirm the hypothesis that severe imbalances within the tightly regulated RAS provoke a broad spectrum of pathologies. Elucidation of the exact pathophysiological roles of the pulmonary RAS certainly contributes to clarification of the strategies used by HCoVs to elicit specific diseases, and might provide a definite demonstration of their etiology. Eventually, a better understanding of HCoV pathogenesis might lead to development of new therapeutic strategies and/or vaccination protocols.

## Future perspective

Deregulation of the RAS may very well be a part of the pathogenicity of HCoVinfections. A therapy aimed at restoring the RAS equilibrium provides the opportunity to treat the symptoms of an infection. Especially in elderly patients, this treatment might be beneficial as the aged population is most vulnerable to deregulation of the RAS. ACE inhibitors and AT_1_ receptor antagonists are in use to decrease high blood pressure. Future research may elucidate the benefit of local administration of these drugs to neutralize the effects of HCoV-NL63- or SARS-CoV-induced ACE2 downregulation in the lungs.

Box 1. **Terminology of renin–angiotensin system-involved aminopeptidases.**

Aminopeptidases constitute a diverse set of peptidases, and serve to proteolytically process amino acid residues from the N-terminus of protein and bioactive peptides. Since these enzymes have been identified using several different characteristics, including number of removed amino acids; residue preference; cellular location; metal ion content and pH at which maximum activity is observed, a particularly complex labyrinth of aminopeptidase classification systems currently exists [116,189]. Aminopeptidase N (EC3.4.11.2) shows a preference for cleavage of neutral amino acid residues. Since Ala is the amino acid most efficiently broken down by this peptidase, it is also named alanyl aminopeptidase. Furthermore, the enzyme is known as microsomal leucine aminopeptidase or aminopeptidase M, reflecting its close association with microsomal membrane fractions in pig kidney from which it was purified [116]. Sequence comparisons revealed that aminopeptidase N is identical to CD13, a cell surface glycoprotein originally defined on subsets of normal and malignant myeloid cells [33]. Aminopeptidase B (EC3.4.11.6) preferentially cleaves N-terminal basic amino acids. Since many enzymatic assays have been performed using Arg-naphthylamide, the enzyme is also known as arginine aminopeptidase. In addition, aminopeptidase B is indicated as arylamidase II or cytosol aminopeptidase IV. Aminopeptidase A (EC3.4.11.7) hydrolyzes N-terminal acidic amino acids. Since glutamyl derivates are most efficiently hydrolyzed, the enzyme is also designated glutamyl aminopeptidase. Alternative names include angiotensinase A, aspartate aminopeptidase, glutamyl peptidase and membrane aminopeptidase II [116].

**Executive summary**

**Coronaviruses are considered important human pathogens**

- Human coronaviruses (HCoVs) initially seemed to be associated primarily with self-limiting upper respiratory tract infections in healthy adults, characterized by mild clinical symptoms.
- SARS-CoV has been proven to cause severe lower respiratory tract infections, causing high morbidity and mortality during the SARS epidemic in 2003.
- At present, all HCoV isolates are being recognized as causative agents of more severe respiratory tract complications, including pneumonia, especially in weakened patients: infants, elderly and immunocompromised individuals.

**Precise pathogenic potential of HCoV species remains largely unconfirmed**

- Since studies needed to unravel a causal link with a specific disease are hampered by a lack of a suitable animal model and/or cell culture system, current knowledge has been primarily obtained through population-based studies.
- Elucidation of the physiologic consequences following virus–host interaction processes might provide alternative insights into the complex process of HCoV pathogenesis.

**The renin–angiotensin system is involved in human coronavirus pathogenesis**

- Two integral proteases of the renin–angiotensin system (RAS) are entry receptors for HCoVs: neutral aminopeptidase and angiotensin-converting enzyme (ACE)2.
- By means of downmodulation of cellular ACE2 expression, SARS-CoV directly impairs normal physiologic function of ACE2 within the RAS. As ACE2 is a prominent negative regulator and protects from worsening of acute lung injury, a loss of ACE2 expression is thought to provoke the severe symptoms observed during infection with SARS-CoV. The potency of HCoV-NL63 to unbalance the RAS needs further investigation.
- Although unconfirmed, abolished cellular expression of aminopeptidase N might prevent over-activation of pulmonary inflammation and organ damage, which might explain the general absence of severe clinical symptoms during HCoV-229E infections.

## References

[1] Gonzalez JM, Gomez-Puertas P, Cavanagh D, Gorbalenya AE, Enjuanes L: A comparative sequence analysis to revise the current taxonomy of the family Coronaviridae. *Arch. Virol.* 148(11) 2207–2235 (2003). 14579179 10.1007/s00705-003-0162-1 PMC7087110

[2] Weiss SR, Navas-Martin S: Coronavirus pathogenesis and the emerging pathogen severe acute respiratory syndrome coronavirus. *Microbiol. Mol. Biol. Rev.* 69(4) 635–664 (2005). 16339739 10.1128/MMBR.69.4.635-664.2005 PMC1306801

[3] Bradburne AF, Bynoe ML, Tyrrell DA: Effects of a ‘new’ human respiratory virus in volunteers. *Br. Med. J.* 3(5568) 767–769 (1967). 6043624 10.1136/bmj.3.5568.767 PMC1843247

[4] vander Hoek L: Human coronaviruses: what do they cause? *Antivir.Ther.* 12(4 Pt B) 651–658 (2007). 17944272

[5] Fouchier RA, Kuiken T, Schutten M *et al.*: Aetiology: Koch’s postulates fulfilled for SARS virus. *Nature* 423(6937) 240 (2003). 12748632 10.1038/423240a PMC7095368

[6] Ksiazek TG, Erdman D, Goldsmith CS *et al.*: A novel coronavirus associated with severe acute respiratory syndrome. *N. Engl. J. Med.* 348(20) 1953–1966 (2003). 12690092 10.1056/NEJMoa030781

[7] Kuiken T, Fouchier RA, Schutten M *et al.*: Newly discovered coronavirus as the primary cause of severe acute respiratory syndrome. *Lancet* 362(9380) 263–270 (2003). 12892955 10.1016/S0140-6736(03)13967-0 PMC7112434

[8] Peiris JS, Lai ST, Poon LL *et al.*: Coronavirus as a possible cause of severe acute respiratory syndrome. *Lancet* 361(9366) 1319–1325 (2003). 12711465 10.1016/S0140-6736(03)13077-2 PMC7112372

[9] Peiris JS, Guan Y, Yuen KY: Severe acute respiratory syndrome. *Nat. Med.* 10(Suppl. 12) S88–S97 (2004). 15577937 10.1038/nm1143 PMC7096017

[10] vander Hoek L, Pyrc K, Jebbink MF *et al.*: Identification of a new human coronavirus. *Nat. Med.* 10(4) 368–373 (2004). 15034574 10.1038/nm1024 PMC7095789

[11] Fouchier RA, Hartwig NG, Bestebroer TM *et al.*: A previously undescribed coronavirus associated with respiratory disease in humans. *Proc. Natl Acad. Sci. USA* 101(16) 6212–6216 (2004). 15073334 10.1073/pnas.0400762101 PMC395948

[12] Woo PC, Lau SK, Chu CM *et al.*: Characterization and complete genome sequence of a novel coronavirus, coronavirus HKU1, from patients with pneumonia. *J. Virol.* 79(2) 884–895 (2005). 15613317 10.1128/JVI.79.2.884-895.2005 PMC538593

[13] Garbino J, Crespo S, Aubert JD *et al.*: A prospective hospital-based study of the clinical impact of non-severe acute respiratory syndrome (non-SARS)-related human coronavirus infection. *Clin. Infect. Dis.* 43(8) 1009–1015 (2006). 16983613 10.1086/507898 PMC7107919

[14] Pyrc K, Berkhout B, van der Hoek L: The novel human coronaviruses NL63 and HKU1. *J. Virol.* 81(7) 3051–3057 (2007). 17079323 10.1128/JVI.01466-06 PMC1866027

[15] Helenius A: Virus entry and uncoating. In: *Field’s Virology* Knipe MD, Howley PM (Eds). Lippincott Williams & Wilkins, PA, USA 99–118 (2007).

[16] Stoddart CA, Geleziunas R, Ferrell S *et al.*: Human immunodeficiency virus type 1 Nef-mediated downregulation of CD4 correlates with Nef enhancement of viral pathogenesis. *J. Virol.* 77(3) 2124–2133 (2003). 12525647 10.1128/JVI.77.3.2124-2133.2003 PMC140869

[17] Michel N, Allespach I, Venzke S, Fackler OT, Keppler OT: The Nef protein of human immunodeficiency virus establishes superinfection immunity by a dual strategy to downregulate cell-surface CCR5 and CD4. *Curr. Biol.* 15(8) 714–723 (2005). 15854903 10.1016/j.cub.2005.02.058

[18] Ross TM, Oran AE, Cullen BR: Inhibition of HIV-1 progeny virion release by cell-surface CD4 is relieved by expression of the viral Nef protein. *Curr. Biol.* 9(12) 613–621 (1999). 10375525 10.1016/s0960-9822(99)80283-8

[19] Aiken C, Konner J, Landau NR, Lenburg ME, Trono D: Nef induces CD4 endocytosis: requirement for a critical dileucine motif in the membrane-proximal CD4 cytoplasmic domain. *Cell* 76(5) 853–864 (1994). 8124721 10.1016/0092-8674(94)90360-3

[20] Schneider-Schaulies J, Schnorr JJ, Brinckmann U *et al.*: Receptor usage and differential downregulation of CD46 by measles virus wild-type and vaccine strains. *Proc. Natl Acad. Sci. USA* 92(9) 3943–3947 (1995). 7732009 10.1073/pnas.92.9.3943 PMC42078

[21] Marschall M, Meier-Ewert H, Herrler G, Zimmer G, Maassab HF: The cell receptor level is reduced during persistent infection with influenza C virus. *Arch. Virol.* 142(6) 1155–1164 (1997). 9229005 10.1007/s007050050149 PMC7087292

[22] Santoro F, Kennedy PE, Locatelli G, Malnati MS, Berger EA, Lusso P: CD46 is a cellular receptor for human herpesvirus 6. *Cell* 99(7) 817–827 (1999). 10619434 10.1016/s0092-8674(00)81678-5

[23] Li W, Moore MJ, Vasilieva N *et al.*: Angiotensin-converting enzyme 2 is a functional receptor for the SARS coronavirus. *Nature* 426(6965) 450–454 (2003). 14647384 10.1038/nature02145 PMC7095016

[24] Kuba K, Imai Y, Rao S *et al.*: A crucial role of angiotensin converting enzyme 2 (ACE2) in SARS coronavirus-induced lung injury. *Nat. Med.* 11(8) 875–879 (2005). 16007097 10.1038/nm1267 PMC7095783

[25] Hofmann H, Pyrc K, van der Hoek L, Geier M, Berkhout B, Pohlmann S: Human coronavirus NL63 employs the severe acute respiratory syndrome coronavirus receptor for cellular entry. *Proc. Natl Acad. Sci. USA* 102(22) 7988–7993 (2005). 15897467 10.1073/pnas.0409465102 PMC1142358

[26] Yeager CL, Ashmun RA, Williams RK *et al.*: Human aminopeptidase N is a receptor for human coronavirus 229E. *Nature* 357(6377) 420–422 (1992). 1350662 10.1038/357420a0 PMC7095410

[27] Gallagher TM, Buchmeier MJ: Coronavirus spike proteins in viral entry and pathogenesis. *Virology* 279(2) 371–374 (2001). 11162792 10.1006/viro.2000.0757 PMC7133764

[28] Marsh M, Helenius A: Virus entry: open sesame. *Cell* 124(4) 729–740 (2006). 16497584 10.1016/j.cell.2006.02.007 PMC7112260

[29] Delmas B, Gelfi J, Sjostrom H, Noren O, Laude H: Further characterization of aminopeptidase-N as a receptor for coronaviruses. *Adv. Exp. Med. Biol.* 342 293–298 (1993). 7911642 10.1007/978-1-4615-2996-5_45

[30] Tresnan DB, Levis R, Holmes KV: Feline aminopeptidase N serves as a receptor for feline, canine, porcine, and human coronaviruses in serogroup I. *J. Virol.* 70(12) 8669–8674 (1996). 8970993 10.1128/jvi.70.12.8669-8674.1996 PMC190961

[31] Li BX, Ge JW, Li YJ: Porcine aminopeptidase N is a functional receptor for the PEDV coronavirus. *Virology* 365(1) 166–172 (2007). 17467767 10.1016/j.virol.2007.03.031 PMC7103304

[32] Lendeckel U, Kahne T, Riemann D, Neubert K, Arndt M, Reinhold D: Review: the role of membrane peptidases in immune functions. *Adv. Exp. Med. Biol.* 477 1–24 (2000). 10849726 10.1007/0-306-46826-3_1

[33] Look AT, Ashmun RA, Shapiro LH, Peiper SC: Human myeloid plasma membrane glycoprotein CD13 (gp150) is identical to aminopeptidase N. *J. Clin. Invest.* 83(4) 1299–1307 (1989). 2564851 10.1172/JCI114015 PMC303821

[34] Sjostrom H, Noren O, Olsen J: Structure and function of aminopeptidase N. *Adv. Exp. Med. Biol.* 477 25–34 (2000). 10849727 10.1007/0-306-46826-3_2

[35] Mina-Osorio P: The moonlighting enzyme CD13: old and new functions to target. *Trends Mol. Med.* 14(8) 361–371 (2008). 18603472 10.1016/j.molmed.2008.06.003 PMC7106361

[36] Riemann D, Kehlen A, Langner J: CD13 – not just a marker in leukemia typing. *Immunol. Today* 20(2) 83–88 (1999). 10098327 10.1016/S0167-5699(98)01398-X PMC7141563

[37] Bhagwat SV, Petrovic N, Okamoto Y, Shapiro LH: The angiogenic regulator CD13/APN is a transcriptional target of Ras signaling pathways in endothelial morphogenesis. *Blood* 101(5) 1818–1826 (2003). 12406907 10.1182/blood-2002-05-1422

[38] Wang G, Deering C, Macke M *et al.*: Human coronavirus 229E infects polarized airway epithelia from the apical surface. *J. Virol.* 74(19) 9234–9239 (2000). 10982370 10.1128/jvi.74.19.9234-9239.2000 PMC102122

[39] Wentworth DE, Tresnan DB, Turner BC *et al.*: Cells of human aminopeptidase N (CD13) transgenic mice are infected by human coronavirus-229E *in vitro*, but not *in vivo* *Virology* 335(2) 185–197 (2005). 15840518 10.1016/j.virol.2005.02.023 PMC7111747

[40] Kolb AF, Hegyi A, Siddell SG: Identification of residues critical for the human coronavirus 229E receptor function of human aminopeptidase N. *J. Gen. Virol.* 78(Pt 11) 2795–2802 (1997). 9367365 10.1099/0022-1317-78-11-2795

[41] Breslin JJ, Mork I, Smith MK *et al.*: Human coronavirus 229E: receptor binding domain and neutralization by soluble receptor at 37°C. *J. Virol.* 77(7) 4435–4438 (2003). 12634402 10.1128/JVI.77.7.4435-4438.2003 PMC150646

[42] Nomura R, Kiyota A, Suzaki E *et al.*: Human coronavirus 229E binds to CD13 in rafts and enters the cell through caveolae. *J. Virol.* 78(16) 8701–8708 (2004). 15280478 10.1128/JVI.78.16.8701-8708.2004 PMC479086

[43] Kawase M, Shirato K, Matsuyama S, Taguchi F: Protease-mediated entry via endosome of human coronavirus 229E. *J. Virol.* 83(2) 712–721 (2008). 18971274 10.1128/JVI.01933-08 PMC2612384

[44] Parton RG, Simons K: The multiple faces of caveolae. *Nat. Rev. Mol. Cell Biol.* 8(3) 185–194 (2007). 17318224 10.1038/nrm2122

[45] Riemann D, Hansen GH, Niels-Christiansen L *et al.*: Caveolae/lipid rafts in fibroblast-like synoviocytes: ectopeptidase-rich membrane microdomains. *Biochem. J.* 354(Pt 1) 47–55 (2001). 11171078 10.1042/0264-6021:3540047 PMC1221627

[46] Delacour D, Jacob R: Apical protein transport. *Cell. Mol. Life Sci.* 63(21) 2491–2505 (2006). 16927027 10.1007/s00018-006-6210-8 PMC11136331

[47] Han DP, Penn-Nicholson A, Cho MW: Identification of critical determinants on ACE2 for SARS-CoV entry and development of a potent entry inhibitor. *Virology* 350(1) 15–25 (2006). 16510163 10.1016/j.virol.2006.01.029 PMC7111894

[48] Tipnis SR, Hooper NM, Hyde R, Karran E, Christie G, Turner AJ: A human homolog of angiotensin-converting enzyme. Cloning and functional expression as a captopril-insensitive carboxypeptidase. *J. Biol. Chem.* 275(43) 33238–33243 (2000). 10924499 10.1074/jbc.M002615200

[49] Donoghue M, Hsieh F, Baronas E *et al.*: A novel angiotensin-converting enzyme-related carboxypeptidase (ACE2) converts angiotensin I to angiotensin 1–9. *Circ. Res.* 87(5) E1–E9 (2000). 10969042 10.1161/01.res.87.5.e1

[50] Vickers C, Hales P, Kaushik V *et al.*: Hydrolysis of biological peptides by human angiotensin-converting enzyme-related carboxypeptidase. *J. Biol. Chem.* 277(17) 14838–14843 (2002). 11815627 10.1074/jbc.M200581200

[51] Guy JL, Lambert DW, Warner FJ, Hooper NM, Turner AJ: Membrane-associated zinc peptidase families: comparing ACE and ACE2. *Biochim. Biophys. Acta* 1751(1) 2–8 (2005). 16054014 10.1016/j.bbapap.2004.10.010 PMC7105243

[52] Nie Y, Wang P, Shi X *et al.*: Highly infectious SARS-CoV pseudotyped virus reveals the cell tropism and its correlation with receptor expression. *Biochem. Biophys. Res. Commun.* 321(4) 994–1000 (2004). 15358126 10.1016/j.bbrc.2004.07.060 PMC7092805

[53] Ren X, Glende J, Al-Falah M *et al.*: Analysis of ACE2 in polarized epithelial cells: surface expression and function as receptor for severe acute respiratory syndrome-associated coronavirus. *J. Gen. Virol.* 87(Pt 6) 1691–1695 (2006). 16690935 10.1099/vir.0.81749-0

[54] Hamming I, Timens W, Bulthuis ML, Lely AT, Navis GJ, van Goor H: Tissue distribution of ACE2 protein, the functional receptor for SARS coronavirus. A first step in understanding SARS pathogenesis. *J. Pathol.* 203(2) 631–637 (2004). 15141377 10.1002/path.1570 PMC7167720

[55] Harmer D, Gilbert M, Borman R, Clark KL: Quantitative mRNA expression profiling of ACE 2, a novel homologue of angiotensin converting enzyme. *FEBS Lett.* 532(1–2) 107–110 (2002). 12459472 10.1016/s0014-5793(02)03640-2

[56] Guo Y, Korteweg C, McNutt MA, Gu J: Pathogenetic mechanisms of severe acute respiratory syndrome. *Virus Res.* 133(1) 4–12 (2008). 17825937 10.1016/j.virusres.2007.01.022 PMC7114157

[57] Oudit GY, Kassiri Z, Jiang C *et al.*: SARS-coronavirus modulation of myocardial ACE2 expression and inflammation in patients with SARS. *Eur. J. Clin. Invest.* 39(7) 618–625 (2009). 19453650 10.1111/j.1365-2362.2009.02153.x PMC7163766

[58] Lambert DW, Yarski M, Warner FJ *et al.*: Tumor necrosis factor-a convertase (ADAM17) mediates regulated ectodomain shedding of the severe-acute respiratory syndrome-coronavirus (SARS-CoV) receptor, angiotensin-converting enzyme-2 (ACE2). *J. Biol. Chem.* 280(34) 30113–30119 (2005). 15983030 10.1074/jbc.M505111200 PMC8062222

[59] Jia HP, Look DC, Tan P *et al.*: Ectodomain shedding of angiotensin converting enzyme 2 in human airway epithelia. *Am. J. Physiol. Lung Cell Mol. Physiol.* 297(1) L84–L96 (2009). 19411314 10.1152/ajplung.00071.2009 PMC2711803

[60] Warner FJ, Lew RA, Smith AI, Lambert DW, Hooper NM, Turner AJ: Angiotensin-converting enzyme 2 (ACE2), but not ACE, is preferentially localized to the apical surface of polarized kidney cells. *J. Biol. Chem.* 280(47) 39353–39362 (2005). 16166094 10.1074/jbc.M508914200

[61] Rice GI, Jones AL, Grant PJ, Carter AM, Turner AJ, Hooper NM: Circulating activities of angiotensin-converting enzyme, its homolog, angiotensin-converting enzyme 2, and neprilysin in a family study. *Hypertension* 48(5) 914–920 (2006). 17000927 10.1161/01.HYP.0000244543.91937.79

[62] Lambert DW, Hooper NM, Turner AJ: Angiotensin-converting enzyme 2 and new insights into the renin–angiotensin system. *Biochem. Pharmacol.* 75(4) 781–786 (2008). 17897633 10.1016/j.bcp.2007.08.012 PMC7111199

[63] Pyrc K, Dijkman R, Deng L *et al.*: Mosaic structure of human coronavirus NL63, one thousand years of evolution. *J. Mol. Biol.* 364(5) 964–973 (2006). 17054987 10.1016/j.jmb.2006.09.074 PMC7094706

[64] Li W, Sui J, HuAng IC *et al.*: The S proteins of human coronavirus NL63 and severe acute respiratory syndrome coronavirus bind overlapping regions of ACE2. *Virology* 367(2) 367–374 (2007). 17631932 10.1016/j.virol.2007.04.035 PMC2693060

[65] Wu K, Li W, Peng G, Li F: Crystal structure of NL63 respiratory coronavirus receptor-binding domain complexed with its human receptor. *Proc. Natl Acad. Sci. USA* 106(47) 19970–19974 (2009). 19901337 10.1073/pnas.0908837106 PMC2785276

[66] Simmons G, Gosalia DN, Rennekamp AJ, Reeves JD, Diamond SL, Bates P: Inhibitors of cathepsin L prevent severe acute respiratory syndrome coronavirus entry. *Proc. Natl Acad. Sci. USA* 102(33) 11876–11881 (2005). 16081529 10.1073/pnas.0505577102 PMC1188015

[67] Huang IC, Bosch BJ, Li F *et al.*: SARS coronavirus, but not human coronavirus NL63, utilizes cathepsin L to infect ACE2-expressing cells. *J. Biol. Chem.* 281(6) 3198–3203 (2006). 16339146 10.1074/jbc.M508381200 PMC8010168

[68] Bosch BJ, Bartelink W, Rottier PJ: Cathepsin L functionally cleaves the severe acute respiratory syndrome coronavirus class I fusion protein upstream of rather than adjacent to the fusion peptide. *J. Virol.* 82(17) 8887–8890 (2008). 18562523 10.1128/JVI.00415-08 PMC2519682

[69] Haga S, Yamamoto N, Nakai-Murakami C *et al.*: Modulation of TNF-α-converting enzyme by the spike protein of SARS-CoV and ACE2 induces TNF-α production and facilitates viral entry. *Proc. Natl Acad. Sci. USA* 105(22) 7809–7814 (2008). 18490652 10.1073/pnas.0711241105 PMC2409424

[70] Glowacka I, Bertram S, Herzog P *et al.*: Differential downregulation of ACE2 by the spike proteins of SARS-coronavirus and human coronavirus NL63. *J. Virol.* 84(2) 1198–1205 (2009). 19864379 10.1128/JVI.01248-09 PMC2798380

[71] Wang S, Guo F, Liu K *et al.*: Endocytosis of the receptor-binding domain of SARS-CoV spike protein together with virus receptor ACE2. *Virus Res.* 136(1–2) 8–15 (2008). 18554741 10.1016/j.virusres.2008.03.004 PMC7114441

[72] Wang H, Yang P, Liu K *et al.*: SARS coronavirus entry into host cells through a novel clathrin- and caveolae-independent endocytic pathway. *Cell Res.* 18(2) 290–301 (2008). 18227861 10.1038/cr.2008.15 PMC7091891

[73] Le Roy C, Wrana JL: Clathrin- and non-clathrin-mediated endocytic regulation of cell signalling. *Nat. Rev. Mol. Cell Biol.* 6(2) 112–126 (2005). 15687999 10.1038/nrm1571

[74] Hansen GH, Delmas B, Besnardeau L *et al.*: The coronavirus transmissible gastroenteritis virus causes infection after receptor-mediated endocytosis and acid-dependent fusion with an intracellular compartment. *J. Virol.* 72(1) 527–534 (1998). 9420255 10.1128/jvi.72.1.527-534.1998 PMC109404

[75] Kurdi M, De Mello WC, Booz GW: Working outside the system: an update on the unconventional behavior of the renin–angiotensin system components. *Int. J. Biochem. Cell Biol.* 37(7) 1357–1367 (2005). 15833268 10.1016/j.biocel.2005.01.012

[76] Zimmerman BG, Dunham EW: Tissue renin–angiotensin system: a site of drug action? *Annu. Rev. Pharmacol. Toxicol.* 37 53–69 (1997). 9131246 10.1146/annurev.pharmtox.37.1.53

[77] Nicholls MG, Richards AM, Agarwal M: The importance of the renin–angiotensin system in cardiovascular disease. *J. Hum. Hypertens.* 12(5) 295–299 (1998). 9655650 10.1038/sj.jhh.1000638

[78] Warner FJ, Lubel JS, McCaughan GW, Angus PW: Liver fibrosis: a balance of ACEs? *Clin. Sci. (Lond.)* 113(3) 109–118 (2007). 17600527 10.1042/CS20070026

[79] Fleming I, Kohlstedt K, Busse R: The tissue renin–angiotensin system and intracellular signalling. *Curr. Opin. Nephrol. Hypertens.* 15(1) 8–13 (2006). 16340660 10.1097/01.mnh.0000196146.65330.ea

[80] Batenburg WW, Jan Danser AH: The (pro)renin receptor: a new addition to the renin–angiotensin system? *Eur. J. Pharmacol.* 585(2–3) 320–324 (2008). 18417113 10.1016/j.ejphar.2008.02.092

[81] Hackenthal E, Paul M, Ganten D, Taugner R: Morphology, physiology, and molecular biology of renin secretion. *Physiol. Rev.* 70(4) 1067–1116 (1990). 2217555 10.1152/physrev.1990.70.4.1067

[82] Paul M, Poyan Mehr A, Kreutz R: Physiology of local renin–angiotensin systems. *Physiol. Rev.* 86(3) 747–803 (2006). 16816138 10.1152/physrev.00036.2005

[83] Unger T, Li J: The role of the renin–angiotensin-aldosterone system in heart failure. *J. Renin Angiotensin Aldosterone Syst.* 5(Suppl. 1) S7–S10 (2004). 15526242 10.3317/jraas.2004.024

[84] Fyhrquist F, Saijonmaa O: Renin–angiotensin system revisited. *J. Intern. Med.* 264(3) 224–236 (2008). 18793332 10.1111/j.1365-2796.2008.01981.x PMC7166930

[85] Lindberg BF, Gyllstedt E, Andersson KE: Conversion of angiotensin I to angiotensin II by chymase activity in human pulmonary membranes. *Peptides* 18(6) 847–853 (1997). 9285934 10.1016/s0196-9781(97)00011-9

[86] Goodfriend TL, Elliott ME, Catt KJ: Angiotensin receptors and their antagonists. *N. Engl. J. Med.* 334(25) 1649–1654 (1996). 8628362 10.1056/NEJM199606203342507

[87] Higuchi S, Ohtsu H, Suzuki H, Shirai H, Frank GD, Eguchi S: Angiotensin II signal transduction through the AT1 receptor: novel insights into mechanisms and pathophysiology. *Clin. Sci. (Lond.)* 112(8) 417–428 (2007). 17346243 10.1042/CS20060342

[88] Suzuki Y, Ruiz-Ortega M, Lorenzo O, Ruperez M, Esteban V, Egido J: Inflammation and angiotensin II. *Int. J. Biochem. Cell Biol.* 35(6) 881–900 (2003). 12676174 10.1016/s1357-2725(02)00271-6

[89] Dragun D, Muller DN, Brasen JH *et al.*: Angiotensin II type 1-receptor activating antibodies in renal-allograft rejection. *N. Engl. J. Med.* 352(6) 558–569 (2005). 15703421 10.1056/NEJMoa035717

[90] Guzik TJ, Hoch NE, Brown KA *et al.*: Role of the T cell in the genesis of angiotensin II induced hypertension and vascular dysfunction. *J. Exp. Med.* 204(10) 2449–2460 (2007). 17875676 10.1084/jem.20070657 PMC2118469

[91] Stegbauer J, Lee DH, Seubert S *et al.*: Role of the renin–angiotensin system in autoimmune inflammation of the central nervous system. *Proc. Natl Acad. Sci. USA* 106(35) 14942–14947 (2009). 19706425 10.1073/pnas.0903602106 PMC2736426

[92] Jones ES, Vinh A, McCarthy CA, Gaspari TA, Widdop RE: AT2 receptors: functional relevance in cardiovascular disease. *Pharmacol. Ther.* 120(3) 292–316 (2008). 18804122 10.1016/j.pharmthera.2008.08.009 PMC7112668

[93] AbdAlla S, Lother H, Abdel-tawab AM, Quitterer U: The angiotensin II AT2 receptor is an AT1 receptor antagonist. *J. Biol. Chem.* 276(43) 39721–39726 (2001). 11507095 10.1074/jbc.M105253200

[94] Li J, Culman J, Hortnagl H *et al.*: Angiotensin AT2 receptor protects against cerebral ischemia-induced neuronal injury. *FASEB J.* 19(6) 617–619 (2005). 15665034 10.1096/fj.04-2960fje

[95] Dinh DT, Frauman AG, Johnston CI, Fabiani ME: Angiotensin receptors: distribution, signalling and function. *Clin. Sci. (Lond.)* 100(5) 481–492 (2001). 11294688

[96] Braun-Menendez E, Fasciolo JC, Leloir LF, Munoz JM: The substance causing renal hypertension. *J. Physiol.* 98(3) 283–298 (1940). 16995204 10.1113/jphysiol.1940.sp003850 PMC1394029

[97] Smith AI, Turner AJ: What’s new in the renin–angiotensin system? *Cell. Mol. Life Sci.* 61(21) 2675–2676 (2004). 15549167 10.1007/s00018-004-4319-1 PMC7079773

[98] Reudelhuber TL: The renin–angiotensin system: peptides and enzymes beyond angiotensin II. *Curr. Opin. Nephrol. Hypertens.* 14(2) 155–159 (2005). 15687842 10.1097/00041552-200503000-00011

[99] Hamming I, Cooper ME, Haagmans BL *et al.*: The emerging role of ACE2 in physiology and disease. *J. Pathol.* 212(1) 1–11 (2007). 17464936 10.1002/path.2162 PMC7167724

[100] Peach MJ: Renin–angiotensin system: biochemistry and mechanisms of action. *Physiol. Rev.* 57(2) 313–370 (1977). 191856 10.1152/physrev.1977.57.2.313

[101] Nicholls MG, Robertson JI: The renin–angiotensin system in the year 2000. *J. Hum. Hypertens.* 14(10–11) 649–666 (2000). 11095157 10.1038/sj.jhh.1001056

[102] Ferrario CM, Trask AJ, Jessup JA: Advances in biochemical and functional roles of angiotensin-converting enzyme 2 and angiotensin-(1–7) in regulation of cardiovascular function. *Am. J. Physiol. Heart Circ. Physiol.* 289(6) H2281–H2290 (2005). 16055515 10.1152/ajpheart.00618.2005 PMC7203566

[103] Danilczyk U, Eriksson U, Crackower MA, Penninger JM: A story of two ACEs. *J. Mol. Med.* 81(4) 227–234 (2003). 12700890 10.1007/s00109-003-0419-x

[104] Rice GI, Thomas DA, Grant PJ, Turner AJ, Hooper NM: Evaluation of angiotensin-converting enzyme (ACE) its homologue ACE2 and neprilysin in angiotensin peptide metabolism. *Biochem. J.* 383(Pt 1) 45–51 (2004). 15283675 10.1042/BJ20040634 PMC1134042

[105] Varagic J, Trask AJ, Jessup JA, Chappell MC, Ferrario CM: New angiotensins. *J. Mol. Med.* 86(6) 663–671 (2008). 18437333 10.1007/s00109-008-0340-4 PMC2713173

[106] Welches WR, Santos RA, Chappell MC, Brosnihan KB, Greene LJ, Ferrario CM: Evidence that prolyl endopeptidase participates in the processing of brain angiotensin. *J. Hypertens.* 9(7) 631–638 (1991). 1653799 10.1097/00004872-199107000-00008

[107] Ferrario CM: Commentary on Tikellis *et al.*: there is more to discover about angiotensin-converting enzyme. *Hypertension* 41(3) 390–391 (2003). 12623932 10.1161/01.HYP.0000060688.57053.7E

[108] Santos RA, Simoes e Silva AC, Maric C *et al.*: Angiotensin-(1–7) is an endogenous ligand for the G protein-coupled receptor Mas. *Proc. Natl Acad. Sci. USA* 100(14) 8258–8263 (2003). 12829792 10.1073/pnas.1432869100 PMC166216

[109] Walters PE, Gaspari TA, Widdop RE: Angiotensin-(1–7) acts as a vasodepressor agent via angiotensin II type 2 receptors in conscious rats. *Hypertension* 45(5) 960–966 (2005). 15767466 10.1161/01.HYP.0000160325.59323.b8

[110] Santos RA, Campagnole-Santos MJ, Andrade SP: Angiotensin-(1–7): an update. *Regul. Pept.* 91(1–3) 45–62 (2000). 10967201 10.1016/s0167-0115(00)00138-5

[111] Santos RA, Ferreira AJ, Simoes ESAC: Recent advances in the angiotensin-converting enzyme 2-angiotensin(1–7)–Mas axis. *Exp. Physiol.* 93(5) 519–527 (2008). 18310257 10.1113/expphysiol.2008.042002

[112] Crackower MA, Sarao R, Oudit GY *et al.*: Angiotensin-converting enzyme 2 is an essential regulator of heart function. *Nature* 417(6891) 822–828 (2002). 12075344 10.1038/nature00786

[113] Gurley SB, Allred A, Le TH *et al.*: Altered blood pressure responses and normal cardiac phenotype in ACE2-null mice. *J. Clin. Invest.* 116(8) 2218–2225 (2006). 16878172 10.1172/JCI16980 PMC1518789

[114] Yamamoto K, Ohishi M, Katsuya T *et al.*: Deletion of angiotensin-converting enzyme 2 accelerates pressure overload-induced cardiac dysfunction by increasing local angiotensin II. *Hypertension* 47(4) 718–726 (2006). 16505206 10.1161/01.HYP.0000205833.89478.5b

[115] Ardaillou R, Chansel D: Synthesis and effects of active fragments of angiotensin II. *Kidney Int.* 52(6) 1458–1468 (1997). 9407491 10.1038/ki.1997.476

[116] Banegas I, Prieto I, Vives F *et al.*: Brain aminopeptidases and hypertension. *J. Renin Angiotensin Aldosterone Syst.* 7(3) 129–134 (2006). 17094048 10.3317/jraas.2006.021

[117] Healy DP, Wilk S: Localization of immunoreactive glutamyl aminopeptidase in rat brain. II. Distribution and correlation with angiotensin II. *Brain Res.* 606(2) 295–303 (1993). 8490722 10.1016/0006-8993(93)90997-2

[118] Harding JW, Yoshida MS, Dilts RP, Woods TM, Wright JW: Cerebroventricular and intravascular metabolism of [^125^I]angiotensins in rat. *J. Neurochem.* 46(4) 1292–1297 (1986). 3950629 10.1111/j.1471-4159.1986.tb00652.x

[119] Ruiz-Ortega M, Lorenzo O, Egido J: Angiotensin III increases MCP-1 and activates NF-κB and AP-1 in cultured mesangial and mononuclear cells. *Kidney Int.* 57(6) 2285–2298 (2000). 10844599 10.1046/j.1523-1755.2000.00089.x

[120] Wright JW, Yamamoto BJ, Harding JW: Angiotensin receptor subtype mediated physiologies and behaviors: new discoveries and clinical targets. *Prog. Neurobiol.* 84(2) 157–181 (2008). 18160199 10.1016/j.pneurobio.2007.10.009 PMC2276843

[121] Ahmad S, Ward PE: Role of aminopeptidase activity in the regulation of the pressor activity of circulating angiotensins. *J. Pharmacol. Exp. Ther.* 252(2) 643–650 (1990). 1968973

[122] Chai SY, Fernando R, Peck G *et al.*: The angiotensin IV/AT4 receptor. *Cell. Mol. Life Sci.* 61(21) 2728–2737 (2004). 15549174 10.1007/s00018-004-4246-1 PMC11924499

[123] Ruiz-Ortega M, Esteban V, Egido J: The regulation of the inflammatory response through nuclear factor-κB pathway by angiotensin IV extends the role of the renin angiotensin system in cardiovascular diseases. *Trends Cardiovasc. Med.* 17(1) 19–25 (2007). 17210474 10.1016/j.tcm.2006.10.003

[124] Mustafa T, Lee JH, Chai SY, Albiston AL, McDowall SG, Mendelsohn FA: Bioactive angiotensin peptides: focus on angiotensin IV. *J. Renin Angiotensin Aldosterone Syst.* 2(4) 205–210 (2001). 11881124 10.3317/jraas.2001.032

[125] Vanderheyden PM: From angiotensin IV binding site to AT(4) receptor. *Mol. Cell Endocrinol.* 302(2) 159–166 (2008). 19071192 10.1016/j.mce.2008.11.015

[126] Esteban V, Ruperez M, Sanchez-Lopez E *et al.*: Angiotensin IV activates the nuclear transcription factor-κB and related proinflammatory genes in vascular smooth muscle cells. *Circ. Res.* 96(9) 965–973 (2005). 10.1161/01.RES.0000166326.91395.74 15831814

[127] Veerappan A, Reid AC, Estephan R *et al.*: Mast cell renin and a local renin–angiotensin system in the airway: role in bronchoconstriction. *Proc. Natl Acad. Sci. USA* 105(4) 1315–1320 (2008). 18202178 10.1073/pnas.0709739105 PMC2234135

[128] Boyce JA: The role of mast cells in asthma. *Prostaglandins Leukot. Essent. Fatty Acids* 69(2–3) 195–205 (2003). 12895603 10.1016/s0952-3278(03)00081-4

[129] Andersson CK, Mori M, Bjermer L, Lofdahl CG, Erjefalt JS: Novel site-specific mast cell subpopulations in the human lung. *Thorax* 64(4) 297–305 (2009). 19131451 10.1136/thx.2008.101683

[130] Campbell DJ, Habener JF: Angiotensinogen gene is expressed and differentially regulated in multiple tissues of the rat. *J. Clin. Invest.* 78(1) 31–39 (1986). 3013940 10.1172/JCI112566 PMC329527

[131] Ohkubo H, Nakayama K, Tanaka T, Nakanishi S: Tissue distribution of rat angiotensinogen mRNA and structural analysis of its heterogeneity. *J. Biol. Chem.* 261(1) 319–323 (1986). 3753601

[132] Studdy PR, Lapworth R, Bird R: Angiotensin-converting enzyme and its clinical significance – a review. *J. Clin. Pathol.* 36(8) 938–947 (1983). 6308066 10.1136/jcp.36.8.938 PMC498427

[133] Bullock GR, Steyaert I, Bilbe G *et al.*: Distribution of type-1 and type-2 angiotensin receptors in the normal human lung and in lungs from patients with chronic obstructive pulmonary disease. *Histochem. Cell Biol.* 115(2) 117–124 (2001). 11444146 10.1007/s004180000235

[134] Wang R, Zagariya A, Ibarra-Sunga O *et al.*: Angiotensin II induces apoptosis in human and rat alveolar epithelial cells. *Am. J. Physiol.* 276(5 Pt 1) L885–L889 (1999). 10330045 10.1152/ajplung.1999.276.5.L885

[135] Papp M, Li X, Zhuang J, Wang R, Uhal BD: Angiotensin receptor subtype AT(1) mediates alveolar epithelial cell apoptosis in response to ANG II. *Am. J. Physiol. Lung Cell Mol. Physiol.* 282(4) L713–L718 (2002). 11880296 10.1152/ajplung.00103.2001

[136] Kuba K, Imai Y, Penninger JM: Angiotensin-converting enzyme 2 in lung diseases. *Curr. Opin. Pharmacol.* 6(3) 271–276 (2006). 16581295 10.1016/j.coph.2006.03.001 PMC7106490

[137] Orte C, Polak JM, Haworth SG, Yacoub MH, Morrell NW: Expression of pulmonary vascular angiotensin-converting enzyme in primary and secondary plexiform pulmonary hypertension. *J. Pathol.* 192(3) 379–384 (2000). 11054722 10.1002/1096-9896(2000)9999:9999<::AID-PATH715>3.0.CO;2-Q

[138] Csaszar A, Halmos B, Palicz T, Szalai C, Romics L: Interpopulation effect of ACE I/D polymorphism on serum concentration of ACE in diagnosis of sarcoidosis. *Lancet* 350(9076) 518 (1997). 10.1016/S0140-6736(05)63108-X 9274601

[139] Specks U, Martin WJ, 2nd, Rohrbach MS: Bronchoalveolar lavage fluid angiotensin-converting enzyme in interstitial lung diseases. *Am. Rev. Respir. Dis.* 141(1) 117–123 (1990). 2153351 10.1164/ajrccm/141.1.117

[140] Idell S, Kueppers F, Lippmann M, Rosen H, Niederman M, Fein A: Angiotensin converting enzyme in bronchoalveolar lavage in ARDS. *Chest* 91(1) 52–56 (1987). 3024928 10.1378/chest.91.1.52

[141] Imai Y, Kuba K, Rao S *et al.*: Angiotensin-converting enzyme 2 protects from severe acute lung failure. *Nature* 436(7047) 112–116 (2005). 16001071 10.1038/nature03712 PMC7094998

[142] Ware LB, Matthay MA: The acute respiratory distress syndrome. *N. Engl. J. Med.* 342(18) 1334–1349 (2000). 10793167 10.1056/NEJM200005043421806

[143] Rubenfeld GD, Caldwell E, Peabody E *et al.*: Incidence and outcomes of acute lung injury. *N. Engl. J. Med.* 353(16) 1685–1693 (2005). 16236739 10.1056/NEJMoa050333

[144] Raiden S, Nahmod K, Nahmod V *et al.*: Nonpeptide antagonists of AT1 receptor for angiotensin II delay the onset of acute respiratory distress syndrome. *J. Pharmacol. Exp. Ther.* 303(1) 45–51 (2002). 12235231 10.1124/jpet.102.037382

[145] Nicholls J, Peiris M: Good ACE, bad ACE do battle in lung injury, SARS. *Nat. Med.* 11(8) 821–822 (2005). 16079870 10.1038/nm0805-821 PMC7095949

[146] Greene LJ, Spadaro AC, Martins AR, Perussi De Jesus WD, Camargo AC: Brain endo-oligopeptidase B: a post-proline cleaving enzyme that inactivates angiotensin I and II. *Hypertension* 4(2) 178–184 (1982). 6175571 10.1161/01.hyp.4.2.178

[147] Marshall RP, Webb S, Bellingan GJ *et al.*: Angiotensin converting enzyme insertion/deletion polymorphism is associated with susceptibility and outcome in acute respiratory distress syndrome. *Am. J. Respir. Crit. Care Med.* 166(5) 646–650 (2002). 12204859 10.1164/rccm.2108086

[148] Jerng JS, Yu CJ, Wang HC, Chen KY, Cheng SL, Yang PC: Polymorphism of the angiotensin-converting enzyme gene affects the outcome of acute respiratory distress syndrome. *Crit. Care Med.* 34(4) 1001–1006 (2006). 16484896 10.1097/01.CCM.0000206107.92476.39

[149] Lew TW, Kwek TK, Tai D *et al.*: Acute respiratory distress syndrome in critically ill patients with severe acute respiratory syndrome. *JAMA* 290(3) 374–380 (2003). 12865379 10.1001/jama.290.3.374

[150] Imai Y, Kuba K, Penninger JM: The discovery of angiotensin-converting enzyme 2 and its role in acute lung injury in mice. *Exp. Physiol.* 93(5) 543–548 (2008). 18448662 10.1113/expphysiol.2007.040048 PMC7197898

[151] Frieman M, Baric R: Mechanisms of severe acute respiratory syndrome pathogenesis and innate immunomodulation. *Microbiol. Mol. Biol. Rev.* 72(4) 672–685 (2008). 19052324 10.1128/MMBR.00015-08 PMC2593566

[152] Chiu RW, Tang NL, Hui DS *et al.*: ACE2 gene polymorphisms do not affect outcome of severe acute respiratory syndrome. *Clin. Chem.* 50(9) 1683–1686 (2004). 15331509 10.1373/clinchem.2004.035436 PMC7108155

[153] Itoyama S, Keicho N, Hijikata M *et al.*: Identification of an alternative 5´-untranslated exon and new polymorphisms of angiotensin-converting enzyme 2 gene: lack of association with SARS in the Vietnamese population. *Am. J. Med. Genet. A* 136(1) 52–57 (2005). 15937940 10.1002/ajmg.a.30779 PMC7138097

[154] Lo AW, Tang NL, To KF: How the SARS coronavirus causes disease: host or organism? *J. Pathol.* 208(2) 142–151 (2006). 16362992 10.1002/path.1897 PMC7168100

[155] Lin HX, Feng Y, Wong G *et al.*: Identification of residues in the receptor-binding domain (RBD) of the spike protein of human coronavirus NL63 that are critical for the RBD-ACE2 receptor interaction. *J. Gen. Virol.* 89(Pt 4) 1015–1024 (2008). 18343844 10.1099/vir.0.83331-0

[156] Mathewson AC, Bishop A, Yao Y *et al.*: Interaction of severe acute respiratory syndrome-coronavirus and NL63 coronavirus spike proteins with angiotensin converting enzyme-2. *J. Gen. Virol.* 89(Pt 11) 2741–2745 (2008). 18931070 10.1099/vir.0.2008/003962-0 PMC2886958

[157] Narayanan K, Huang C, Makino S: SARS coronavirus accessory proteins. *Virus Res.* 133(1) 113–121 (2008). 18045721 10.1016/j.virusres.2007.10.009 PMC2720074

[158] Weidmann P, De Myttenaere-Bursztein S, Maxwell MH, de Lima J: Effect on aging on plasma renin and aldosterone in normal man. *Kidney Int.* 8(5) 325–333 (1975). 538 10.1038/ki.1975.120

[159] Corman B, Barrault MB, Klingler C *et al.*: Renin gene expression in the aging kidney: effect of sodium restriction. *Mech. Ageing Dev.* 84(1) 1–13 (1995). 8719773 10.1016/0047-6374(95)01630-i

[160] Nicholls JM, Peiris JS, Chan KH, Poon LM, Beh SL: Occult respiratory viral infections in coronial autopsies: a pilot project. *Hong Kong Med. J.* 15(3) S13–15 (2009). 19509431

[161] Bastien N, Anderson K, Hart L *et al.*: Human coronavirus NL63 infection in Canada. *J. Infect. Dis.* 191(4) 503–506 (2005). 15655772 10.1086/426869 PMC7199484

[162] Cameron MJ, Bermejo-Martin JF, Danesh A, Muller MP, Kelvin DJ: Human immunopathogenesis of severe acute respiratory syndrome (SARS). *Virus Res.* 133(1) 13–19 (2008). 17374415 10.1016/j.virusres.2007.02.014 PMC7114310

[163] Myint S, Johnston S, Sanderson G, Simpson H: Evaluation of nested polymerase chain methods for the detection of human coronaviruses 229E and OC43. *Mol. Cell Probes* 8(5) 357–364 (1994). 7877631 10.1006/mcpr.1994.1052 PMC7135046

[164] Arbour N, Day R, Newcombe J, Talbot PJ: Neuroinvasion by human respiratory coronaviruses. *J. Virol.* 74(19) 8913–8921 (2000). 10982334 10.1128/jvi.74.19.8913-8921.2000 PMC102086

[165] Arbour N, Ekande S, Cote G *et al.*: Persistent infection of human oligodendrocytic and neuroglial cell lines by human coronavirus 229E. *J. Virol.* 73(4) 3326–3337 (1999). 10074187 10.1128/jvi.73.4.3326-3337.1999 PMC104097

[166] Reaux A, de Mota N, Zini S *et al.*: PC18, a specific aminopeptidase N inhibitor, induces vasopressin release by increasing the half-life of brain angiotensin III. *Neuroendocrinology* 69(5) 370–376 (1999). 10343178 10.1159/000054439

[167] Ruiz-Ortega M, Lorenzo O, Ruperez M *et al.*: Role of the renin–angiotensin system in vascular diseases: expanding the field. *Hypertension* 38(6) 1382–1387 (2001). 11751722 10.1161/hy1201.100589

[168] Wolf G: Renal injury due to renin–angiotensin-aldosterone system activation of the transforming growth factor-β pathway. *Kidney Int.* 70(11) 1914–1919 (2006). 16985515 10.1038/sj.ki.5001846

[169] Ruiz-Ortega M, Lorenzo O, Egido J: Angiotensin III up-regulates genes involved in kidney damage in mesangial cells and renal interstitial fibroblasts. *Kidney Int. Suppl.* 68 S41–S45 (1998). 9839282 10.1046/j.1523-1755.1998.06811.x

[170] Border WA, Noble NA: Transforming growth factor b in tissue fibrosis. *N. Engl. J. Med.* 331(19) 1286–1292 (1994). 7935686 10.1056/NEJM199411103311907

[171] Marshall RP, McAnulty RJ, Laurent GJ: Angiotensin II is mitogenic for human lung fibroblasts via activation of the type 1 receptor. *Am. J. Respir. Crit. Care Med.* 161(6) 1999–2004 (2000). 10852780 10.1164/ajrccm.161.6.9907004

[172] Li X, Molina-Molina M, Abdul-Hafez A, Uhal V, Xaubet A, Uhal BD: Angiotensin converting enzyme-2 is protective but downregulated in human and experimental lung fibrosis. *Am. J. Physiol. Lung Cell Mol. Physiol.* 295(1) L178–L185 (2008). 18441099 10.1152/ajplung.00009.2008 PMC2494775

[173] Uhal BD, Kim JK, Li X, Molina-Molina M: Angiotensin-TGF-β1 crosstalk in human idiopathic pulmonary fibrosis: autocrine mechanisms in myofibroblasts and macrophages. *Curr. Pharm. Des.* 13(12) 1247–1256 (2007). 17504233 10.2174/138161207780618885

[174] Weber KT: Fibrosis, a common pathway to organ failure: angiotensin II and tissue repair. *Semin. Nephrol.* 17(5) 467–491 (1997). 9316215

[175] Wynn TA: Cellular and molecular mechanisms of fibrosis. *J. Pathol.* 214(2) 199–210 (2008). 18161745 10.1002/path.2277 PMC2693329

[176] Thomas WG, Mendelsohn FA: Angiotensin receptors: form and function and distribution. *Int. J. Biochem. Cell Biol.* 35(6) 774–779 (2003). 12676163 10.1016/s1357-2725(02)00263-7

[177] Li YD, Block ER, Patel JM: Activation of multiple signaling modules is critical in angiotensin IV-induced lung endothelial cell proliferation. *Am. J. Physiol. Lung Cell Mol. Physiol.* 283(4) L707–L716 (2002). 12225947 10.1152/ajplung.00024.2002

[178] Patel JM, Martens JR, Li YD, Gelband CH, Raizada MK, Block ER: Angiotensin IV receptor-mediated activation of lung endothelial NOS is associated with vasorelaxation. *Am. J. Physiol.* 275(6 Pt 1) L1061–L1068 (1998). 9843842 10.1152/ajplung.1998.275.6.L1061

[179] Rangel R, Sun Y, Guzman-Rojas L *et al.*: Impaired angiogenesis in aminopeptidase N-null mice. *Proc. Natl Acad. Sci. USA* 104(11) 4588–4593 (2007). 17360568 10.1073/pnas.0611653104 PMC1815469

[180] Danziger RS: Aminopeptidase N in arterial hypertension. *Heart Fail. Rev.* 13(3) 293–298 (2008). 18008160 10.1007/s10741-007-9061-y PMC7088157

[181] Kuhlmann UC, Chwieralski CE, van den Brule S *et al.*: Modulation of cytokine production and silica-induced lung fibrosis by inhibitors of aminopeptidase N and of dipeptidyl peptidase-IV-related proteases. *Life Sci.* 84(1–2) 1–11 (2008). 18973761 10.1016/j.lfs.2008.10.001

[182] Tani K, Ogushi F, Huang L *et al.*: CD13/aminopeptidase N, a novel chemoattractant for T lymphocytes in pulmonary sarcoidosis. *Am. J. Respir. Crit. Care Med.* 161(5) 1636–1642 (2000). 10806168 10.1164/ajrccm.161.5.9902008

[183] Heikkinen T, Jarvinen A: The common cold. *Lancet* 361(9351) 51–59 (2003). 12517470 10.1016/S0140-6736(03)12162-9 PMC7112468

[184] Turner RB, Weingand KW, Yeh CH, Leedy DW: Association between interleukin-8 concentration in nasal secretions and severity of symptoms of experimental rhinovirus colds. *Clin. Infect. Dis.* 26(4) 840–846 (1998). 9564459 10.1086/513922

[185] Zhu Z, Tang W, Ray A *et al.*: Rhinovirus stimulation of interleukin-6 *in vivo* and *in vitro* Evidence for nuclear factor κB-dependent transcriptional activation. *J. Clin. Invest.* 97(2) 421–430 (1996). 8567963 10.1172/JCI118431 PMC507033

[186] Perlman S, Dandekar AA: Immunopathogenesis of coronavirus infections: implications for SARS. *Nat. Rev. Immunol.* 5(12) 917–927 (2005). 16322745 10.1038/nri1732 PMC7097326

[187] Colten HR, Krause JE: Pulmonary inflammation – a balancing act. *N. Engl. J. Med.* 336(15) 1094–1096 (1997). 9091809 10.1056/NEJM199704103361511

[188] Cole JM, Khokhlova N, Sutliff RL *et al.*: Mice lacking endothelial ACE: normal blood pressure with elevated angiotensin II. *Hypertension* 41(2) 313–321 (2003). 12574101 10.1161/01.hyp.0000050650.52007.83

[189] Taylor A: Aminopeptidases: structure and function. *FASEB J.* 7(2) 290–298 (1993). 8440407 10.1096/fasebj.7.2.8440407

